# Shiga Toxin-Producing Escherichia coli-Associated Hemolytic Uremic Syndrome Following Neoadjuvant Chemotherapy for Advanced Ovarian Cancer: A Case Report

**DOI:** 10.7759/cureus.106251

**Published:** 2026-04-01

**Authors:** Ratko Delić

**Affiliations:** 1 Obstetrics and Gynaecology, Celje General Hospital, Celje, SVN

**Keywords:** acute kidney injury, advanced ovarian cancer, hemolytic uremic syndrome (hus), neoadjuvant chemotherapy, shiga toxin-producing escherichia coli

## Abstract

Hemolytic uremic syndrome (HUS) is an uncommon but serious cause of thrombotic microangiopathy (TMA) in adults and can pose diagnostic challenges, especially in patients receiving chemotherapy, due to overlapping features with chemotherapy-induced TMA.

We report the case of a 55-year-old woman with advanced ovarian cancer undergoing neoadjuvant chemotherapy who developed Shiga toxin-producing *Escherichia coli* (STEC)-associated HUS. The patient developed severe oligo-anuric acute kidney injury (AKI), requiring hemodialysis and multiple transfusions. Early recognition, prompt supportive care, and multidisciplinary management were critical for stabilization and safe continuation of oncologic therapy. This case highlights the importance of considering STEC-HUS in immunocompromised adults presenting with TMA during oncologic chemotherapy.

## Introduction

Hemolytic uremic syndrome (HUS) is a thrombotic microangiopathy (TMA) characterized by microangiopathic hemolytic anemia (MAHA), thrombocytopenia, and acute kidney injury (AKI). It is traditionally classified as typical HUS, most commonly associated with Shiga toxin-producing *Escherichia coli* (STEC-HUS), and atypical HUS, which is usually related to complement dysregulation [1,2]. In addition, secondary forms associated with drugs, malignancy, pregnancy, or systemic diseases have also been described [1,2].

Adults with malignancy, particularly women with ovarian cancer, are a vulnerable population. Immunosuppression from the underlying disease, chemotherapy, and nutritional deficiencies increases the risk of infections, severe complications, and intensive care unit admission.

STEC-HUS in ovarian cancer patients is rare and can be difficult to distinguish from chemotherapy-induced TMA due to overlapping clinical and laboratory features. Recognizing this distinction early is critical for initiating appropriate supportive care and multidisciplinary management, which can optimize outcomes while allowing continuation of oncologic therapy.

## Case presentation

**Figure 1:**
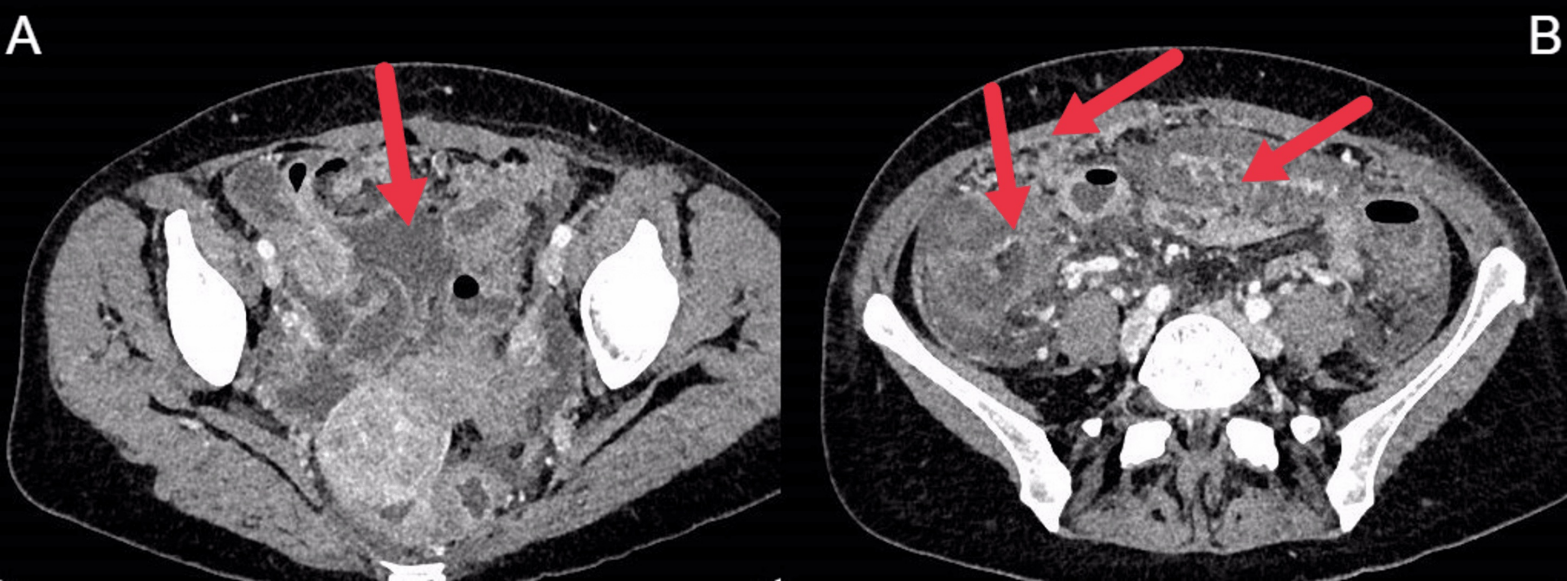
High-resolution abdominal CT images showing ascites (A, arrow) and an enlarged right ovary, omental infiltration, and bowel wall thickening with marked submucosal edema (B, arrows). CT: computed tomography

A 55-year-old woman with advanced ovarian cancer was hospitalized after the first cycle of neoadjuvant chemotherapy with paclitaxel and carboplatin, presenting with acute abdominal pain and bloody diarrhea. One month earlier, diagnostic laparoscopy had revealed advanced, inoperable, high-grade serous ovarian cancer, classified as stage IIIC according to the International Federation of Gynecology and Obstetrics (FIGO) system. During hospitalization, laboratory tests were regularly monitored. An abdominal computed tomography (CT) scan revealed ascites with peritoneal deposits, an enlarged right ovary, omental caking, and circumferential bowel wall thickening with marked submucosal edema (Figure 1). Multiplex polymerase chain reaction (PCR) testing of a stool sample (FilmArray gastrointestinal panel; BioFire Diagnostics, Salt Lake City, Utah, USA) detected STEC, and schistocytes were observed on the peripheral blood smear.

Before transfer to the Nephrology Department, the patient received aggressive intravenous fluid therapy. Despite this, she developed oliguria. Laboratory evaluation demonstrated markedly elevated inflammatory markers and evidence of AKI, with increased creatinine and blood urea nitrogen (BUN) levels. Thrombocytopenia and markedly elevated lactate dehydrogenase (LDH) were also observed. Key laboratory findings are summarized in Table 1.

**Table 1:** Laboratory findings before transfer to the Nephrology Department.

Parameter	Result	Reference Range
C-reactive protein (CRP)	82.6 mg/L	< 5 mg/L
Leukocytes	18.4 × 10^9^/L	4.0-10.0 × 10^9^/L
Hemoglobin	125 g/L	120-150 g/L
Thrombocytes	114 × 10^9^/L	150-450 × 10^9^/L
Creatinine	250 μmol/L	44-80 μmol/L
Blood urea nitrogen (BUN)	16.3 mmol/L	1.7-8.3 mmol/L
Lactate dehydrogenase (LDH)	18.81 μkat/L	< 4.13 μkat/L
Aspartate aminotransferase (AST)	0.83 μkat/L	< 0.52 μkat/L
Alanine aminotransferase (ALT)	0.52 μkat/L	< 0.56 μkat/L
Gamma-glutamyl transferase (GGT)	1.00 μkat/L	< 0.63 μkat/L
Schistocytes	0.5%	< 0.1%
Differential leukocyte count (DLC)	Neutrophils 78%, Lymphocytes 5%, Monocytes 6%	Neutrophils 40-75%, Lymphocytes 20-50%, Monocytes <10%
Sodium (Na)	127.7 mmol/L	135.0-145.0 mmol/L
Potassium (K)	4.32 mmol/L	3.80-5.50 mmol/L
Chloride (Cl)	99.9 mmol/L	95.0-105.0 mmol/L

The patient was hospitalized in the Nephrology Department for one month, during which she underwent hemodialysis and received nine units of packed red blood cells, albumin, and platelet transfusions. During this period, fever and hypotension developed, and antibiotic therapy with ciprofloxacin and metronidazole was initiated following positive blood cultures for *Enterobacter cloacae* complex. She was eventually discharged in stable condition.

After stabilization of renal function, neoadjuvant chemotherapy was resumed as carboplatin monotherapy, based on a multidisciplinary decision by the gynecologic oncology team in coordination with nephrologists. Laboratory tests and renal function were monitored prior to each carboplatin cycle.

Cytoreductive surgery, including abdominal hysterectomy, bilateral adnexectomy, and omentectomy, was subsequently performed. All surgical specimens confirmed infiltration by high-grade serous carcinoma cells. Shortly after surgery, and following postoperative carboplatin monotherapy, the patient developed an ileus requiring additional surgery and formation of an ileostomy. Despite these interventions, she died several months later due to disease progression.

## Discussion

Typical HUS is characterized by the simultaneous presence of MAHA, thrombocytopenia, and AKI [1,2]. It is most commonly associated with STEC infection, particularly the enterohemorrhagic O157:H7 serotype, and represents a systemic disorder driven by toxin-mediated endothelial injury and a generalized inflammatory response [2].

While STEC-HUS predominantly affects children, cases in adult oncology patients are exceedingly rare, making early recognition and individualized supportive care essential for optimal outcomes [3,4]. Complications following STEC infection in immunocompromised adults are associated with high morbidity and mortality, likely because a lower infectious inoculum may be sufficient to cause severe disease in these subgroups [5]. Patients with advanced ovarian cancer undergoing chemotherapy represent a particularly vulnerable population, as immunosuppression, mucosal barrier injury, and treatment-related cytopenias may increase susceptibility to severe STEC-HUS. Early recognition of STEC-HUS, often in patients with preexisting organ injury, is vital to improving prognosis and reducing mortality and long-term complications [6].

Recognized risk factors for STEC infection include ingestion of contaminated food or water, close contact with infected individuals, and exposure to animals, with domestic cattle representing the main reservoir for the O157:H7 serotype [7]. Notably, local sanitary inspection identified contamination of the patient’s residential water supply around the time of STEC infection. To our knowledge, only this patient developed severe STEC-HUS, highlighting the critical role of host factors, particularly immunocompromised status, in determining disease severity. In this case, the combination of clinical features (hemorrhagic colitis, abdominal pain, and oliguria), laboratory findings (MAHA, schistocytes, thrombocytopenia, elevated BUN, and LDH), and detection of STEC in stool enabled the early diagnosis of STEC-HUS.

The kidney is the organ most frequently affected in STEC-HUS, with approximately half of patients requiring dialysis, as illustrated by the severe oligo-anuric acute AKI in our patient [8]. While the kidney is the primary target, STEC-HUS is a multisystem disease that can affect virtually any organ. Gastrointestinal manifestations may include hemorrhagic colitis (as seen in our patient), bowel ischemia or necrosis, and, in severe cases, perforation [8]. Diagnosing hemorrhagic colitis in patients undergoing chemotherapy is particularly challenging, as gastrointestinal bleeding may result from multiple causes, including chemotherapy-induced mucosal injury. Neurological involvement in STEC-HUS can be sudden and severe, representing the most frequent cause of acute mortality, and may manifest as altered mental status, stroke, seizures, or coma [9].

Beyond renal, gastrointestinal, and neurological involvement, extrarenal complications described in the literature include pancreatitis, with pancreatic beta-cell dysfunction leading to insulin-dependent diabetes mellitus; cardiac microvascular TMA, with elevated troponin levels; and rhabdomyolysis, among others [8]. Importantly, these manifestations may arise not only during the acute phase but also after apparent clinical recovery.

Supportive care remains the cornerstone of therapy, including fluid and electrolyte management, renal replacement therapy when indicated, transfusions of blood products, and careful hemodynamic monitoring [10]. Antibiotic therapy is generally discouraged in STEC-HUS because certain antibiotics may increase Shiga toxin release, potentially worsening the disease.

In our patient, ciprofloxacin and metronidazole were administered due to positive blood cultures with *E. cloacae* complex, representing a true systemic infection [6]. Following one month of intensive supportive care, renal function stabilized, allowing resumption of neoadjuvant chemotherapy. Given the recent episode of STEC-HUS and AKI, continuation of combination chemotherapy was considered high-risk. Carboplatin monotherapy was therefore chosen by a multidisciplinary team to balance renal safety with continuation of oncologic treatment, representing a justified, individualized approach in this high-risk patient. Although chemotherapy-induced TMA has been reported with various antineoplastic agents, including platinum-based drugs used in combination regimens, specific evidence implicating carboplatin or paclitaxel alone is limited [11,12].

The temporal relationship in this case, together with classical STEC-HUS laboratory and clinical findings, strongly supports Shiga toxin-mediated endothelial injury as the primary cause. Nevertheless, the overlapping clinical features emphasize the need for careful differential diagnosis in oncology patients presenting with TMA, as early recognition is essential for guiding supportive care and for planning safe continuation or modification of oncologic treatment [13].

## Conclusions

Adult oncology patients are particularly vulnerable to STEC-HUS, and early recognition is critical to prevent severe complications. Clinicians must carefully distinguish STEC-HUS from chemotherapy-induced TMA, as these conditions can present with overlapping features. Multidisciplinary management is essential to optimize patient outcomes, particularly in immunocompromised individuals, whose disease severity may be disproportionately affected, even when environmental exposures are similar to others. This case represents a rare occurrence of STEC-HUS in an adult oncology patient, contributing to the existing literature by highlighting diagnostic challenges and management considerations in this vulnerable population.
